# Leaching Behavior of Heavy Metals and Transformation of Their Speciation in Polluted Soil Receiving Simulated Acid Rain

**DOI:** 10.1371/journal.pone.0049664

**Published:** 2012-11-21

**Authors:** Shun-an Zheng, Xiangqun Zheng, Chun Chen

**Affiliations:** 1 Agro-Environmental Protection Institute, Ministry of Agriculture, Tianjin, People’s Republic of China; 2 Key Laboratory of Production Environment and Agro-Product Safety, Ministry of Agriculture, Tianjin, People’s Republic of China; 3 Tianjin Key Laboratory of Agro-Environment and Agro-Product Safety, Tianjin, People’s Republic of China; University of Kansas, United States of America

## Abstract

Heavy metals that leach from contaminated soils under acid rain are of increasing concern. In this study, simulated acid rain (SAR) was pumped through columns of artificially contaminated purple soil. Column leaching tests and sequential extraction were conducted for the heavy metals Cu, Pb, Cd, and Zn to determine the extent of their leaching as well as to examine the transformation of their speciation in the artificially contaminated soil columns. Results showed that the maximum leachate concentrations of Cu, Pb, Cd, and Zn were less than those specified in the Chinese Quality Standards for Groundwater (Grade IV), thereby suggesting that the heavy metals that leached from the polluted purple soil receiving acid rain may not pose as risks to water quality. Most of the Pb and Cd leachate concentrations were below their detection limits. By contrast, higher Cu and Zn leachate concentrations were found because they were released by the soil in larger amounts as compared with those of Pb and Cd. The differences in the Cu and Zn leachate concentrations between the controls (SAR at pH 5.6) and the treatments (SAR at pH 3.0 and 4.5) were significant. Similar trends were observed in the total leached amounts of Cu and Zn. The proportions of Cu, Pb, Cd, and Zn in the EXC and OX fractions were generally increased after the leaching experiment at three pH levels, whereas those of the RES, OM, and CAR fractions were slightly decreased. Acid rain favors the leaching of heavy metals from the contaminated purple soil and makes the heavy metal fractions become more labile. Moreover, a pH decrease from 5.6 to 3.0 significantly enhanced such effects.

## Introduction

Acid rain has been a well-known environmental problem for decades and can lead to acidification of surface waters and soils. Acid deposition is formed from SO_2_ and NO_x_ emitted to the atmosphere, largely because of fossil-fuel combustion. The most important sources are energy production, especially coal- and oil-fired power plants, and transportation sources, such as vehicles and ships. The air pollutants are transformed in the atmosphere to H_2_SO_4_ and HNO_3_, transported across distances potentially as far as hundreds of kilometers, and deposited as precipitation (wet deposition) and as gas and particles (dry deposition) [1], [2]. Acid deposition is also an environmental problem of increasing concern in China, where acid rain is mainly distributed in the areas of Yangtze River to the south, Qinghai-Tibet Plateau to the east, and in the Sichuan Basin. About 40% of the total territory of China is affected by the acid rain [3]. Sichuan Basin is one of the most severely hit-area of acid rain in southern China, where the rapid industrialization for last few decades has caused fast growth in sulfur emissions. Based on the monitoring data for 21 cities in Sichuan Province within the State-Controlled-Network of China, the number of cities with the annual average pH value of acid rain lower than 5.6 was 19. According to the environmental protection and monitoring agencies in Sichuan Province, the direct economic loss due to acid rain is estimated to be U. S. $ 3 billion for one year [4].

Sichuan basin is also known as the “Red Basin” because it is mainly covered by red or purple rock series of the Trias–Cretaceous system, from which the purple soils, one of the most important soils for agricultural production in subtropical areas of China, are developed and formed. Purple soil, classified as Eutric Regosols in FAO Taxonomy or Pup-Calric-Entisol in the Chinese soil taxonomy, is typically characterized by thin soil horizons and inherited many of the characteristics of parent materials or rocks, such as its color ranging from purple to red. But other changes in purple soil properties have taken place as a result of land use changes, agricultural practices, or eco-environment disturbances [5], [6]. Due to the soil background and human activities, the soil has been severely contaminated by heavy metals (commonly including Cu, Pb, Cd and Zn) in many areas, resulting in potential risk to local human health and environment [7], [8].

There is accumulating evidence that acid rain is able to enhance metal mobilization in soil ecosystems. Previous studies demonstrated that the H^+^ ion in the acidic water displaces the cations from their binding sites, reduces the cation exchange capacity (CEC), and increases the concentrations of these cations in the soil-water system. The negatively charged sulfate and nitrate ions in the acid rain can act as “counter-ions”, which allow cations to be leached from the soil. Through a series of chemical reactions, cations such as K^+^, Na^+^, Ca^2+^ and Mg^2+^ are leached out and become unavailable to plants as nutrients [9], [10]. Likewise, toxic ions, such as Cu, Pb and Cd, usually bound to the negatively charged surface of soil particles can be displaced by H^+^ ion too [11].

Leaching is the process by which contaminants are transferred from a stabilized matrix to liquid medium, such as water or other solutions. However, the influence of acid rain on the leaching behavior of heavy metals and transformation of their speciation in polluted purple soil is not investigated in detail. The objectives of this study were to evaluate the leaching of heavy metals Cu, Pb, Cd and Zn in a contaminated purple soil affected by simulated acid rain (SAR) over a range of pH, and to identify how simulated acid rain influences the chemical speciation of these metals in purple soil. Column experiments were used in this study to provide information about element release, transport in soil, and chemistry of soil and leachates.

## Materials and Methods

### Soil Sample Collection and Analysis

The analyzed soil sample was collected from a 0 cm to 20 cm layer of agricultural purple soil in the Pengzhou Agro-ecological Station of the Chinese Academy of Agricultural Sciences in Sichuan Province, China. The study area has a subtropical humid monsoon climate with an average annual precipitation of 850 mm to 1000 mm and an average annual temperature of 15°C to 16°C. The soil samples were air-dried, ground, and passed through a 2 mm sieve. Selected soil characteristics determined by standard methods [12] were 6.27 for pH, 0.64 g·kg^−1^ for CaCO_3_, 26.51 g·kg^−1^ for organic matter, 20.81 g·kg^−1^ for CEC, 265.41 g·kg^−1^ for clay (<0.002 mm), 51.84 mg·kg^−1^ for total copper, 43.92 mg·kg^−1^ for total lead, 0.36 mg·kg^−1^ for total cadmium, 121.23 mg·kg^−1^ for total zinc.

### Contaminated Soil Preparation

Artificially contaminated soils were composed of the collected purple soil. The load quantities of Cu [Cu(NO_3_)_2_·3H_2_O], Pb [Pb(NO_3_)_2_], Cd [Cd(NO_3_)_2_·4H_2_O], and Zn [Zn(NO_3_)_2_·6H_2_O] were as follows: Cu+Pb+Cd+Zn = 400 mg·kg^–1^+500 mg·kg^–1^+1 mg·kg^–1^+500 mg·kg^–1^. Based on the levels of polluted soil defined by the Chinese Environmental Protection Agency, the selected concentrations in this study represented moderately contaminated soils (Grade III of the National Soil Heavy Metals Standards GB15618-1995). The thoroughly mixed soil samples were stored and incubated at a 75% water holding capacity for 12 mon. The chemical speciation of aged soil was then determined by the sequential extraction procedure of Tessier et al. [13]. The chemical reagents, extraction conditions, and their corresponding fractions are defined as follows:

1. Exchangeable fraction (EXC): 2 g of the soil sample (oven-dry weight), 16 mL 1.0 mol·L^−1^ MgCl_2_, pH 7, shake vigorously in a reciprocating shaker for 1 h, 20°C.
2. Carbonate-bound fraction (CAR): 16 mL of pH 5, 1.0 mol·L^−1^ sodium acetate, shake vigorously in the reciprocating shaker for 5 h, 20°C.
3. The Fe/Mn oxide-bound fraction (OX): 40 mL of 0.04 mol·L^−1^ NH_4_OH·HCl in 25% (v/v) acetic acid at pH 3 for 5 h at 96°C with occasional agitation.
4. The OM-bound fraction: 6 mL of 0.02 mol·L^−1^ HNO_3_ and 10 mL of 30% H_2_O_2_ (pH adjusted to 2 with HNO_3_), water bath, 85°C for 5 h with occasional agitation. 10 mL of 3.2 mol·L^−1^ NH_4_OAc in 20% (v/v) HNO_3_, shake vigorously in the reciprocating shaker for 30 min.
5. Residual fraction (RES): Dried in a force-air oven at 40°C, 24 h. Subsamples after sieving with 0.149 mm openings were used for determining Cu, Zn, Pb and Cd contents.

Extractions were performed in 100 mL polypropylene centrifuge tubes. Between each successive extraction, the supernatant was centrifuged at 1500×*g* for 30 min and then filtered using a membrane filter (0.45-µm nominal pore size).

### Simulated Acid Rain Preparation

Simulated acid rain was designed according to the main ion composition and pH of the local rain water (the pH of rainfall varied from 3.0 to 5.6) [4]. Synthetic acid rain with pH values of 3.0, 4.5, and 5.6 was prepared from a stock H_2_SO_4_–HNO_3_ solution (4∶1, v/v). The concentrations of Ca^2+^, NH_4_^+^, Mg^2+^, 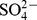, 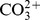, Cl^–^, and K^+^ in SAR were 1.5, 2.62, 1.00, 10.00, 2.61, 11.17, and 1.78 mg·L^–1^, respectively.

### Leaching Experiment

**Figure 1:**
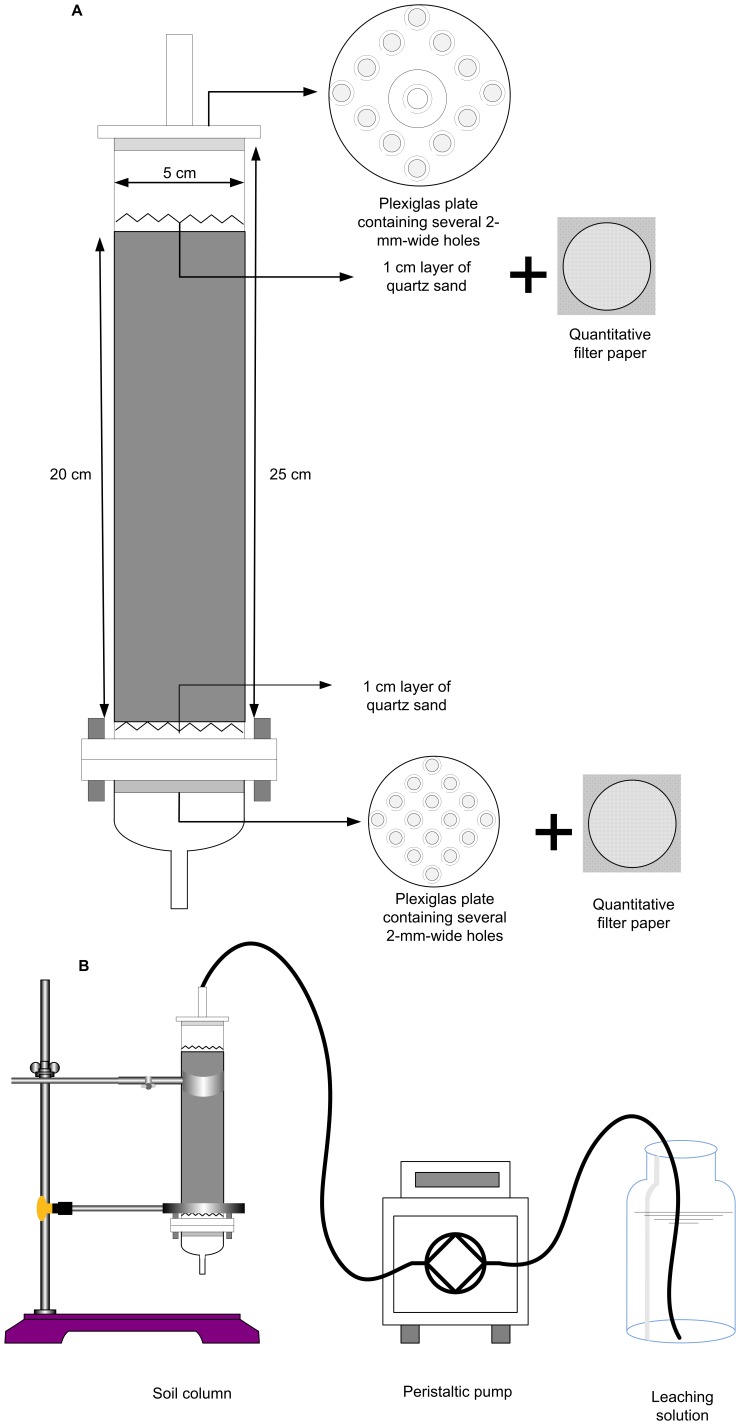
Leaching experimental design. (A) Schematic diagram of soil column. (B) Schematic analytical setup for the measurement of metal concentrations in the leaching experiment.

The column (Fig. 1A) was oriented vertically and slowly saturated from the bottom with deionized water until it reached the field-holding capacity. The soil column was allowed to stabilize for 24 h. The feed solutions were composed of SAR at pH 3.0, 4.5, and 5.6, with the last group as the control. The feed solutions were then introduced into the system using a peristaltic pump to percolate through the packed soil columns at a flow rate of (60±5) mL·h^–1^, which corresponds to the field infiltration velocity of 3.0 cm·h^–1^ [14] (Fig. 1B). The redox potential was measured in one-third of the columns to check the aeration of columns and to avoid waterlogging conditions. Each column was flushed with 3000 mL (1530 mm) of the incoming solution, which corresponds to 1.5 yr of precipitation (rain) in the study area. Experiments were conducted in triplicate at each pH treatment.

The leachate from the soil column was filtered through a 0.45 µm membrane filter, and 200 mL of the filtered leachate (equivalent to 100 mm of added SAR) was sampled using glass collectors to determine the heavy metal concentrations, pH, and electrical conductivity. After the tests, the columns were separated and extruded. All of the soils from the entire depth range were thoroughly mixed, dried, and ground to analyze the chemical speciation of the heavy metal residues using the sequential extraction procedure [13].

### Metal Determination and Quality Control

The Cu, Zn, Pb, and Cd concentrations in soil were determined by digesting 0.5 g of the soil samples (oven-dry weight) with a HNO_3_–HF–HClO_4_ mixture followed by elemental analysis. The concentrations of these metals in all the solutions were analyzed by graphite furnace atomic absorption spectrometry (AA220Z; Varian, USA). The detection limits for Cu, Zn, Pb, and Cd were 2, 2, 1, and 0.5 µg·L^–1^, respectively. All the reagents used for analysis were of analytical grade or higher. All the containers were soaked in 10% HCl, rinsed thoroughly in deionized water, and dried before use. The standard substances such as the geochemical standard reference sample soil in China (GSS-15) were used to examine the precision and accuracy of determination. The relative errors (REs) between the sum of the metal concentration in individual fractions and the measured total metal concentration in the soil samples, which ranged from –12.36% to 9.44%, were calculated to check the reliability of the sequential extraction procedure.

### Statistical Analysis

Data were analyzed using the Origin 8.5.1 for Windows software at the 5% and 1% significance levels.

## Results and Discussion

### Heavy Metal Concentrations in the Leachate

The Pb and Cd concentrations in the leachate from SAR treatments at pH 3.0, 4.5, and 5.6 were generally very low throughout the leaching period, with most of them below their detection limits (data not shown). The maximum Pb and Cd concentrations in the leachate were only (17.0±2.16) and (2.8±0.42) µg·L^–1^, respectively, which are both less than the limits of the Chinese drinking water quality standards (50 and 10 µg·L^–1^, respectively). No significant differences were observed among the treatments of SAR at different pH levels. This result suggested that the polluted purple soil, which received SAR at a given pH in our study, may not cause groundwater contamination by Pb and Cd leaching. Low leachate concentrations of Pb and Cd can probably be attributed to the low mobility of Pb in soil as well as the comparatively low levels of Cd in artificially contaminated purple soil [15], [16].

**Figure 2:**
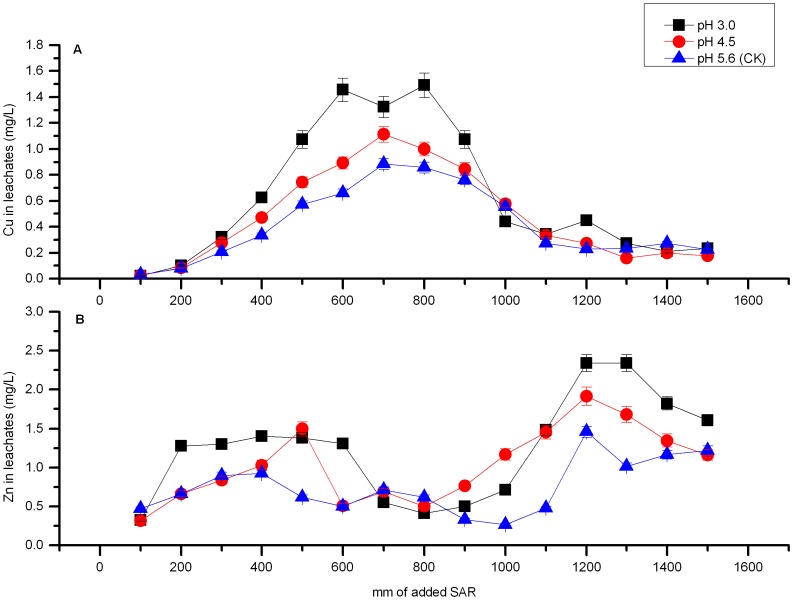
Metal concentrations in leachates as functions of addition of SAR. The concentrations of Cu and Zn in the leachates as functions of addition of SAR at different pHs. (A) Copper. (B) Zinc.

The Different results were observed with Cu and Zn. The leaching concentrations of Cu and Zn under SAR conditions are shown in Fig. 2. Higher Cu and Zn leachate concentrations were observed throughout the entire leaching period (ranging from 0.021 mg·L^–1^ to 1.49 mg·L^–1^ for Cu; 0.019 mg·L^–1^ to 2.34 mg·L^–1^ for Zn) as compared with those of Pb and Cd. However, the maximum Cu and Zn concentrations were still below the Chinese Quality Standards for Groundwater (Grade IV), thereby suggesting that leaching from the polluted purple soil under acid rain at the pH used in this study is unlikely to cause Cu and Zn contamination in water systems.

Fig. 2A shows the Cu concentrations in the leachates when SAR was used as the leaching reagent. Similar trends were observed for the changes in the Cu concentration of each treatment. The application of SAR produced a pulse of Cu in the leachates during the first stage of the experiment, with peaks of 1.49, 1.11, and 0.88 mg·L^–1^ at a pH of 3.0, 4.5, and 5.6, respectively. The concentrations decreased thereafter and reached <0.2 mg·L^–1^ at the end of the experiment. A small amount of Cu (<0.18 mg·L^−1^) was leached from the soil column in the first 200 mm of rainfall, which may be attributed to the required time for Cu to move from the upper surface layer to the bottom of the soil column and for the Cu-bound compounds to functionalize with the soil components. The SAR treatments at pH 3.0 and 4.5 yielded higher Cu concentrations than those in the control (SAR at pH 5.6), particularly before 1000 mm of SAR was added. The Cu leachate concentration increased as the pH values of SAR decreased. However, the Cu leachate concentrations for the treatments at pH 3.0 and 4.5 were approximately equal to those in the control as the leaching amounts were increased, thereby suggesting that the leachable Cu in all of the treatments was decreased. After 1200 mm of SAR was added, no significant differences were observed between the control and the treatments at pH 3.0 and 4.5.

The changes in the Zn leachate concentrations against the leaching amounts in the SAR treatments at different pH levels are shown in Fig. 2B. The variation curves of Zn with the three treatments were generally similar. Two peak values of the Zn concentration were found during the leaching experiment. The first peak appeared after approximately 300 mm to 500 mm of continuous leaching, and the second one appeared after 1200 mm of SAR was added. The peak values of the Zn concentration in the leachates were 2.34, 1.92, and 1.46 mg·L^–1^ for the treatments at pH 3.0, 4.5, and 5.6, respectively. These results suggested that the release of Zn from the contaminated purple soil can be observed in two stages. In the first leaching stage, leached Zn mainly existed as water soluble and exchangeable fractions. After being leached out, these mobile Zn began to adsorb and desorb in the soil column with the downward movement of leachate, and after 300–500 mm rainfall Zn concentration in the leachate reached the first peak value. During the second stage, Zn was released from different fractions such as the CAR-, OX-, and OM-bound Cu. A comparable amount of Zn was shifted from those fractions, which revealed the second peak values after 1200 mm of rainfall was added. Many studies have demonstrated that Zn in EXC and the water soluble fraction usually account for only 0.5% to 7% of the total Zn concentration [17]–[19], which could explain why the second peak values were higher than the first ones.

### Total Leaching Amounts of Heavy Metals

An analysis of the total leaching amounts of heavy metals can directly reflect the leaching strength of the said heavy metals. The total leaching amounts of Pb and Cd were not calculated because most of their concentrations in the leachate were below the detection limits. The total leaching amounts of Cu and Zn in each treatment are shown in Table 1. Two-way ANOVA showed that the total amounts of the leached metals from the soil columns were significantly affected by the pH level and metal species as well as the interaction between these two factors (Table 2). Zn leached more easily from the soil column than Cu at each pH level (Tables 1 and 2). Similarly, Guo et al. [20] revealed that more Zn flowed out of the soil column than Cu in acidic soils under SAR treatments. The amount of leached metals increased at pH 3.0 and 4.5 as compared with that in the controls (pH 5.6), whereas the magnitude of this increase depended on the metal. Metallic elements become more soluble (leachable) under acidic conditions [21]. pH may likewise control the nature of the interactions between metals and soil surfaces [22]. Thus, higher Cu and Zn concentrations were induced by SAR and dissolved in columns at lower pH (Table 1). The H^+^ ion in acid rain displaces the cations from their binding sites, which causes the increased amount of heavy metal desorption, as shown in the following reactions [Eq. (1–2)]:
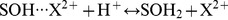
(1)

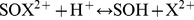
(2)


**Table 1:** Total amounts of the metals leached from the soil columns in mg of metal per kg of soil.

Treatment	Total leaching amounts (mg·kg^−1^)	
	Cu	Zn
pH 3.0	(3.93±0.44) a	(7.81±0.61) a
pH 4.5	(2.98±0.36) b	(6.47±0.47) b
pH 5.6	(2.27±0.39) c	(4.72±0.33) c

Note: The values are means ± standard deviation. Different lower case letters show significant differences in the same treatment (ANOVA/LSD, *P*<0.05).

**Table 2:** Two-way ANOVA of pH levels and metal species effects for total amounts of the metals leached from the soil columns.

Source of variance	SS	MS	F
pH	48.22	48.22	245.75**
Metal species	16.93	8.46	43.14**
pH × metal species	1.64	0.82	4.18*
Error	2.35	0.20	
Total	69.14		

Note: **significant at 99% probability level, *significant at 95% probability level.

The different affinities of the binding sites to metals may cause the different rates and amounts of the desorbed metal. For Zn, greater amounts were released (Table 1). H^+^ ions can only replace some metal cations, whereas the surface OH^–^ groups attached to the Fe or Al atoms can accept protons from the solution, thereby increasing the positive surface charges via the protonation of mineral surfaces [23], [24]. Protonation is a very rapid reaction during the initial stage and depends on the amounts and properties of ferric oxides.
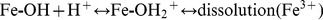
(3)


The surface protonation in soil promotes the dissolution of ferric oxides in acidic solutions [Eq. (3)], which exhibit high adsorptive capacities for heavy metals and consequently increase the solubilization of these metals in their oxide fractions.

### Chemical Speciation of Heavy Metals in Purple Soil Affected by SAR

**Figure 3:**
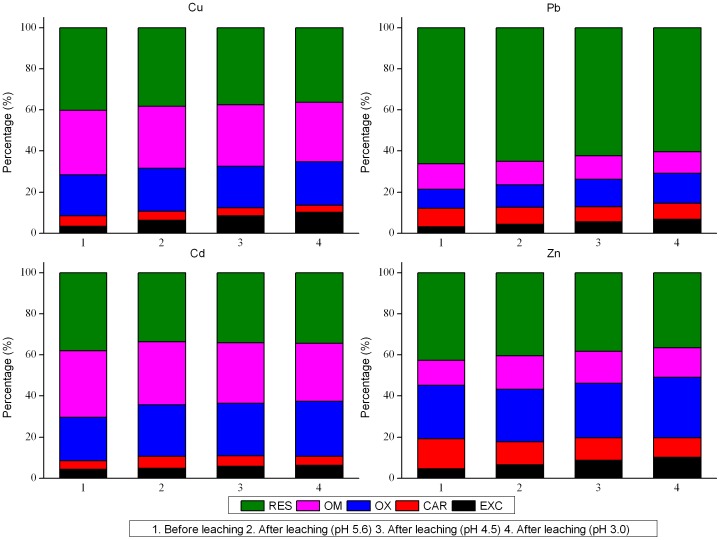
Variation of the percentage of metals in different fractions. Species distribution of Cu, Pb, Cd and Zn extracted with Tessier scheme before and after the column tests.

The chemical fractions of Cu, Pb, Cd, and Zn in soils before and after the column leaching tests are shown in Fig. 3. For the original contaminated purple soil, Cu and Cd were dominantly associated with RES (37% to 41%), followed by OM (31% to 33%). Generally, EXC- and CAR-bound Cu and Cd accounted for <9% of the total amount of the respective metals. A significant fraction of Pb in the soils was bound in RES (66.31%), and four fractions accounted for <35% of the total Pb. Some studies have shown [15], [25] that Pb is mostly present in the RES of soil at the surface or profile scale and is widely considered to exhibit very low geochemical mobility. The amount of Zn in soils was mainly associated with RES (42.69%), then with the OX (25.96%), followed by the CAR (14.44%), and OM (12.23%). The percent of exchangeable Zn in the soils was relatively low (4.69%).

Some variations in the proportions of the four metals in five fractions after the tests are shown by Fig. 3. The relative concentrations of the elements in the EXC and OX fractions were generally increased after the leaching experiment was conducted for the three treatments, whereas those of RES, OM, and CAR fractions were slightly decreased. The Pb and Cd concentrations in EXC ranged from 3.28% to 4.39% and 4.47% to 6.79%, respectively. By contrast, these metals increased by more than 3% in OX. The increase in the Cu concentrations in EXC was greater than that in the OX (EXC 3% to 7% vs. OX <2%). The relative concentrations of Zn in OM and OX significantly increased, particularly in the EXC fraction (average 4.32%).

The EXC fraction was usually the first to be brought into the solution and is readily available for plant uptake. The four trace elements in EXC, with high bioavailability and mobility, were increased from ∼1.2% to 5.03%, which indicated the increased direct risk of Cu, Pb, Cd, and Zn contamination in the soil/groundwater system as caused by acid rain. The amounts of the non-residual fractions represent the amounts of potentially active trace elements [25]. Generally, the high proportion of trace elements in the non-residual fractions of soils may suggest the large contributions of anthropogenic elements. The non-residual fractions of Cu, Pb, Cd, and Zn in the soil zones after the leaching tests averaged at 62.55%, 37.43%, 65.95%, and 61.61%, respectively. The non-residual fractions of Cu, Pb, Cd, and Zn in the original soil samples averaged at 59.75%, 33.69%, 62.04%, and 57.31%, respectively. The increased non-residual fractions represented the increased potential risk of Cu, Pb, Cd, and Zn contamination in the soil/groundwater system. Simple correlation analysis indicated that the pH was significantly correlated with the exchangeable heavy metal content (*r* = 0.974, *P*<0.01). Likewise, the pH was significantly correlated with the non-residual heavy metal content (*r* = 0.968, *P*<0.01). These data suggested that acid rain favors the leaching of heavy metals from the contaminated purple soil. Consequently, acid rain can affect the mobilization potential of heavy metals. The decrease in the pH from 5.6 to 3.0 significantly enhanced such effects of acid rain, which are likely to cause more serious harmful effects on soil-vegetation systems.

### Conclusions

The maximum leachate concentrations of Cu, Pb, Cd, and Zn from the soil that received SAR were less than those in the Chinese Quality Standard for Groundwater (Grade IV). Moreover, most of the Pb and Cd leachate concentrations were below their detection limits. By contrast, higher Cu and Zn leachate concentrations were observed because these metals were released in higher amounts as compared with Pb and Cd. The differences in the Cu and Zn leachate concentrations between the control (SAR at pH 5.6) and the treatments (SAR at pH 3.0 and 4.5) were significant. Similar trends were observed in the total leaching amounts of Cu and Zn. The proportions of Cu, Pb, Cd, and Zn in the EXC and OX fractions were generally increased after the leaching experiment at three pH levels, whereas those of the RES, OM, and CAR fractions were slightly decreased. Acid rain favors the leaching of heavy metals from the contaminated purple soil and causes the heavy metal fractions to become more labile. A decrease in the pH from 5.6 to 3.0 significantly enhanced such effects. However, the experiment is only an indoor simulation that used artificially contaminated purple soil. Thus, long-term field experiments on soils contaminated with acid rain should be conducted to study the cycling effect of heavy metal residues in the soil or those that leach into the groundwater.
